# Adverse outcome pathways: Application to enhance mechanistic understanding of neurotoxicity

**DOI:** 10.1016/j.pharmthera.2017.05.006

**Published:** 2017-11

**Authors:** Anna Bal-Price, M.E. (Bette) Meek

**Affiliations:** a European Commission Joint Research Centre, Directorate F – Health, Consumers and Reference Materials, Ispra, Italy; b McLaughlin Centre for Risk Science, University of Ottawa, Ottawa, Canada

**Keywords:** AD, Alzheimer disease, ADME, absorption, distribution, metabolism and excretion, AO, adverse outcome, AOP, adverse outcome pathway, AOP-KB, adverse outcome pathway knowledge base, BDNF, brain derived neurotrophic factor, B/H, Bradford-Hill, CI, complex I of mitochondrial chain, CNS, Central nervous system, DNT, developmental neurotoxicity, HTT, high-throughput toxicity testing, IATA, integrated approaches to testing and assessment, KE, key event, KER, key event relationship, MIE, molecular initiating event, MOA, mode of action, MPTP, 1-methyl-4-phenyl-3104 1,2,3,6-tetrahydropyridine, NT, neurotoxicity, OECD, Organisation for Economic Co-operation and Development, PBPK, physiologically based pharmacokinetic, PD, Parkinson's disease, QSAR, quantitative structure activity relationship, TFHA, Task Force for Hazard Assessment, WNT, Working Group of the National Coordinators for the Test Guidelines Programme, WOE, weight of evidence, Key event, Key event relationship, Weight of evidence, Quantitation, Neurotoxicity, Mechanistic understanding

## Abstract

Recent developments have prompted the transition of empirically based testing of late stage toxicity in animals for a range of different endpoints including neurotoxicity to more efficient and predictive mechanistically based approaches with greater emphasis on measurable key events early in the progression of disease. The adverse outcome pathway (AOP) has been proposed as a simplified organizational construct to contribute to this transition by linking molecular initiating events and earlier (more predictive) key events at lower levels of biological organization to disease outcomes. As such, AOPs are anticipated to facilitate the compilation of information to increase mechanistic understanding of pathophysiological pathways that are responsible for human disease.

In this review, the sequence of key events resulting in adverse outcome (AO) defined as parkinsonian motor impairment and learning and memory deficit in children, triggered by exposure to environmental chemicals has been briefly described using the AOP framework. These AOPs follow convention adopted in an Organization for Economic Cooperation and Development (OECD) AOP development program, publically available, to permit tailored application of AOPs for a range of different purposes.

Due to the complexity of disease pathways, including neurodegenerative disorders, a specific symptom of the disease (e.g. parkinsonian motor deficit) is considered as the AO in a developed AOP. Though the description is necessarily limited by the extent of current knowledge, additional characterization of involved pathways through description of related AOPs interlinked into networks for the same disease has potential to contribute to more holistic and mechanistic understanding of the pathophysiological pathways involved, possibly leading to the mechanism-based reclassification of diseases, thus facilitating more personalized treatment.

## 1 Introduction to AOPs

Traditionally, assessment of hazard associated with exposure of the general population to chemicals in the occupational and ambient environments has been based on empirical evidence of late stage effects based on a defined series of individual apical endpoints in animals exposed to high doses. This necessitates conduct of a range of relatively costly short and long term studies to address endpoints individually (e.g., neurotoxicity, cancer, reproductive and developmental toxicity) and high dose (often over many orders of magnitude) and species (rodent to human) extrapolations as a basis for comparison with exposure to estimate risk to humans. Focus on more mechanistically based approaches in which early markers on the path to toxicity are examined in human cell-based in vitro models at more realistic doses has potential to be much more predictive, decreasing the costs of testing and reducing the uncertainty associated with current interspecies and dose extrapolations.

Recent regulatory pressures and technological advances are prompting a shift to these potentially more efficient and predictive mechanistically based approaches. These include increasing regulatory requirements worldwide to more efficiently consider the hazard and risk associated with the thousands of chemicals in commerce introduced prior to requirement for assessment under modern chemicals legislation (EC, 2017b, EPA, 2016, Meek and Armstrong, 2007, NICNAS, 2017) and mandated requirements to reduce or eliminate the use of animals in toxicity testing of drugs and chemicals (including cosmetics) (EC, 2017a). Concomitant advances in technology to measure effects at much lower levels of biological organization in high throughput platforms also have potential to contribute to the development and application of mechanistic data in designing more efficient and effective testing strategies.

As a consequence of these developments, new strategies have been proposed to facilitate transition to more mechanistically based approaches. The U.S. National Research Council report “Toxicity testing in the 21st Century: A Vision and A Strategy” published in 2007 proposed a shift in focus from high dose testing to identify late stage events in animals to largely in vitro testing of early, predictive key events at human relevant doses in human cell –based models with subsequent quantitative in vitro to in vivo extrapolation (Krewski et al., 2010).

Transition to these more predictive approaches necessitates the assimilation and assessment of mechanistic data to better characterize biological systems in formalized descriptions. The Adverse Outcome Pathway (AOP), which evolved from the ecotoxicological community as a means to enhance the utility of predictive tools such as quantitative structure activity relationship (QSAR), biomarkers, and other types of mechanistic data for both understanding and predicting potential adverse effects of chemical exposure in wildlife populations provides a helpful, simplifying and coordinating construct in this transition (Ankley et al., 2010, Edwards et al., 2016).

**Fig. 1:**
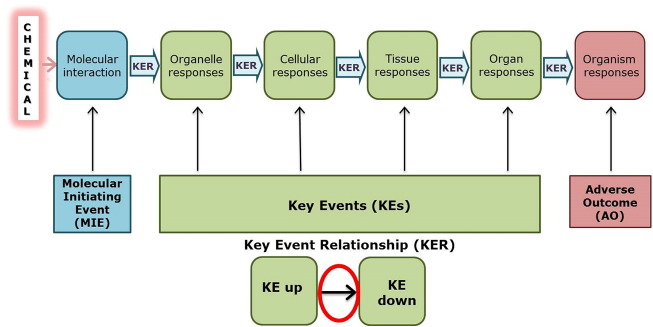
An AOP consists of key events (KEs) and key events relationships (KERs) at different levels of biological organization starting from an initial interaction of a chemical with the biological system (molecular initiating event; MIE) through a sequence of KEs (cellular, tissue, organ and organism) leading to an adverse outcome (AO) of regulatory relevance that represents overt adversity at either organism or population level. At sufficient concentrations and durations of exposure, KE up will trigger KE down, overcoming cell defence mechanisms and adaptation processes.

AOPs are based on the premise that toxicity is the result of biological motifs of failure initiated by the interaction of a chemical with biomolecules involved in critical physiological pathways (Ankley et al., 2010). Conceptually, an AOP can be viewed, then, as a sequence of key events commencing with the initial interaction of a stressor with a biomolecule in a target cell or tissue (i.e., the molecular initiating event), progressing through a dependent series of intermediate key events at different levels of biological organization (cell, tissue, organ), culminating in an adverse outcome defined currently by regulatory requirements (e.g., neurotoxicity) in either individuals or populations (Fig. 1).

AOPs are represented as linear pathways anchored by specialized key events (KEs), namely a Molecular Initiating Event (MIE), representing the direct interaction of a chemical with a biological target and an adverse outcome (AO) at the organism (e.g. disease or overt toxicity) or population level (i.e. inability to maintain a particular species in its native habitat). The adverse outcome is one considered to be of regulatory significance – e.g., the types of late stage outcomes such as cancer that have traditionally been measured in mammalian toxicity studies. Remaining intermediate KEs represent the most important steps in a plausibly hypothesized pathway leading from the molecular initiating event to the adverse outcome (Fig. 1); they must be empirically observable (i.e., reproducibly measurable) and necessary to trigger the outcome (i.e., the AO). Empirical evidence is based on relevant studies described in the literature or results from experiments specifically designed for the purpose of AOP development. The adverse outcomes can vary considerably, including, for example, the wide range of endpoints measured in traditional mammalian toxicity studies (e.g., cancer, neurotoxicity, immunotoxicity, etc.).

By design, then, AOPs are simplified representations of disease pathways with key events at various levels of biological organization which inform purpose specific application in regulatory context. AOPs are not intended to provide full mechanistic molecular descriptions of causality. Illustrated as a linear series of key events, in reality AOPs represent interdependent networks of events with feedback loops in which disease outcomes are initiated or modified.

Chemically agnostic (i.e., biological pathways that can be initiated by a range of chemicals that can trigger the MIE), simplified, currently linear descriptions of pathways between MIEs and AOs described by AOPs are anticipated to contribute, then, to characterization of networks in systems biology, through sharing of KEs and key event relationships (KERs). They are also anticipated to contribute to shifting focus from the late stage effects (apical endpoints) observed in traditional toxicity testing to molecular initiating and early key events as a basis to develop mechanistically based and more predictive markers for adverse effects or disease. KERs (i.e., defining the structural and functional relationship between a pair of KEs) are also a critical focus of AOPs, representing their largely quantitative predictive element, framing essential components for quantitative predictive disease model design and development (Wittwehr et al., 2017).

## 2 Documenting AOPs and considering scientific confidence in the supporting data

Recognizing the importance of accessing and coordinating AOP development and assessment to contribute to this transition, in 2012, the OECD launched a Development Program http://www.oecd.org/chemicalsafety/testing/adverse-outcome-pathways-molecular-screening-and-toxicogenomics.htm with subsequent release, in 2013, of initial guidance for the description of AOPs (OECD, 2013) and a subsequent Handbook (OECD, 2016b), which outlines best practices for defining, documenting and assessing AOPs in an associated AOP knowledge base (AOP-KB), based on accumulating experience. The AOP knowledge base (AOP-KB) which serves as a single source for generated AOPs, is a critical element of engagement and communication in the OECD program, integrating both research and regulatory input. Specifically, it encourages collaboration in the generation and description of AOPs of researchers working at different levels of biological organization. It also facilitates the sharing of KEs and KERs among multiple AOPs, as a basis for assembling networks (contributing to descriptions of systems biology). In addition to improving the consistency in reporting, the structured description and assessment of developed AOPs for the knowledge base is anticipated to increase common understanding of the nature of often critical and recurring data gaps to meaningfully inform potential regulatory application.

Systematic and transparent assessment of the extent of supporting data for AOPs or weight of evidence (WOE) is an important aspect of their inclusion in the (AOP-KB) with the objective of facilitating their potential utility and application in regulatory context. WOE for hypothesized AOPs draws upon a subset of the Bradford Hill (B/H) considerations (Meek, Palermo, Bachman, North, & Jeffrey Lewis, 2014) initially proposed to assess the causality in epidemiological studies but modified to address the objectives of mode of action (MOA) analysis and evolved recently based on increasing experience (Meek et al., 2014a, Meek et al., 2014b).

The subset of chemically independent B/H considerations relevant for AOPs includes biological plausibility, essentiality of KEs and empirical support of KERs. These considerations (in rank order of their considered importance) are defined and distinguished as follows: (i) biological plausibility of KERs relates to how well we understand the mechanistic structural/functional relationships of the pathway. In essence, do we know enough to be able to “predict” what happens if the pathway is disturbed (experimentally)?; (ii) essentiality of KEs relates to experimental support for whether or not downstream KEs or the AO are prevented or modified if an upstream event is blocked (for example, testing in knockout models or investigations of reversibility); (iii) available empirical data should support the patterns of temporal and dose-response relationships between and among KEs induced by stressors (reference chemicals) which are known to impact the pathway.

An Annex to the OECD Handbook defines these considerations and provides guidance on examples of the nature of the data for high, moderate and low confidence determinations (Becker et al., 2015, OECD, 2016b). Supporting templates are also provided, to guide the developer or assessor in making these calls. The objective is to provide a consistent and communicable measure of the extent of the supporting data and associated confidence based on clearly delineated rationales, and to identify inconsistencies, uncertainties and critical data gaps relevant to considered confidence for different types of regulatory application.

## 3 Application of MOA/AOP including mechanistically based toxicity testing

Organization of mechanistic knowledge at a range of different levels of biological organization to facilitate its compilation, integration and evaluation for research and regulatory application in the AOP construct is conceptually similar to that for mode of action (MOA) which has long been considered in the application of mechanistic data in risk assessment of specific chemicals or chemical groups. MOA is defined as a “biologically plausible series of key events leading to an adverse effect” (Sonich-Mullin et al., 2001). Frameworks for the systematic consideration of the weight of supporting evidence for MOA in animals and subsequently, relevance to humans taking into account qualitative and quantitative differences have long been developed and adopted in international and national guidance and assessments. They continue to evolve to take into consideration advancements in emerging technologies such as high-throughput toxicity testing (HTT) (Meek, Boobis, et al., 2014).

However, AOPs vary subtly from MOA in that their focus is on predominantly predictive aspects i.e., the extent to which molecular initiating and early KEs can inform progression to adversity of regulatory significance. Similarly to AOPs they are considered as the series of KEs, initiated by an interaction of the chemical with the molecular target (protein, DNA) triggering the biological pathway to disease (adverse outcome). MOA, on the other hand, has always been considered on a chemical or group specific basis and takes into account aspects of toxicokinetics and metabolism, not addressed by AOPs.

Mechanistic information has long been considered for application in human health risk assessment, as a basis to determine MOA in hazard characterization and resulting implications for subsequent dose-response analysis in hazard characterization. Envisaged application of AOPs in a more predictive context necessarily varies as a function of the extent of supporting data and quantitation of KERs. Potential applications are envisaged to range from chemicals grouping for read-across to priority setting for testing and assessment to fully quantitative predictive characterization of hazard and risk assessment for specific chemicals. It can be performed based on computational models, taking into account chemical specific toxicokinetic and metabolic aspects (Meek, Boobis, et al., 2014).

Appropriate regulatory application of AOPs requires explicit consideration of the degree of confidence required in the extent of the supporting evidence (i.e., qualitative weight of evidence), the need to characterize interconnected networks and the required degree of quantification of KERs. Indeed, the varied nature of potential applications is the motivating basis for structured descriptions of AOPs in the knowledge base, with identified critical data gaps, delineation of the extent of confidence in qualitative supporting data and the extent of quantitation of the KERs.

**Fig. 2:**
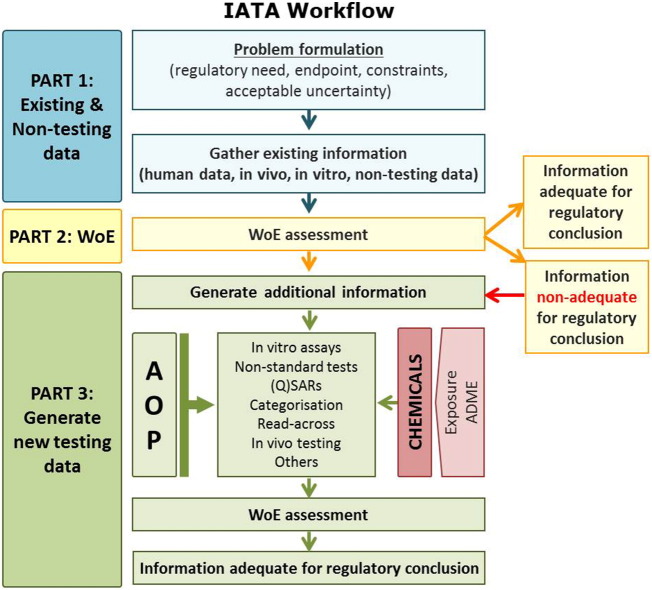
An integrated Approach to Testing and Assessment (IATA) designed for fit-for-purpose consideration of testing strategies integrates multiple sources of existing information (human data, in vivo, in vitro and non-testing data) and guides the targeted generation of new data when required. The degree to which IATA needs to be complemented by information as delineated in the associated AOP is dependent on the decision context.

In relation to transition of toxicity testing, AOPs are considered to provide a helpful ‘working hypothesis’ for data weighting and integration in more targeted and efficient testing strategies, commonly referenced as IATA (integrated approaches to testing and assessment) (Worth & Patlewicz, 2016). IATA encompasses an iterative “fit-for-purpose” approach to address a defined question of hazard, safety or risk assessment within a specific regulatory decision context (Tollefsen et al., 2014). The weight of evidence from multiple sources of information, including that from different levels of biological organization, obtained by a variety of non-test and test methods [(Q)SAR, read-across, in chemico, in vitro, ex vivo, in vivo] or omic technologies (e.g. toxicogenomics) is considered (Tollefsen et al., 2014). This is assessed initially to determine whether it is sufficient to address the regulatory decision under consideration, taking into account the purpose-specific degree of uncertainty. Alternatively, if the information is inadequate, the AOP assists in the identification of the nature and extent of new data that should be generated (Fig. 2). For example, the AOP framework provides the biological context for inclusion of the results of in vitro assays at lower levels of biological organization that could be selected for inclusion in a battery of tests. An advanced example involves the evaluation of assays monitoring the KEs from the skin sensitization AOP (Reisinger et al., 2015). Similarly, early KEs could potentially identify early, predictive biomarkers of human AO (disease).

As such, IATA plays a pivotal role in shifting emphasis from testing to identify hazard based on a range of defined endpoints and protocols in traditional toxicity testing in animals to more tailored, thoughtful and predictive approaches taking into account existing mechanistic data at various levels of biological organization.

A practical application of AOP-informed IATA for a specific chemical or group of chemicals will depend on the decision context (chemical screening and prioritization, hazard or risk assessment). These considerations will influence the construction of IATA in terms of different data requirements, types of testing (e.g. in vitro, in chemico, in vivo), non-testing methods (QSAR, read-across), data integration approaches and acceptable level of uncertainty.

## 4 Challenges in developing AOPs relevant to developmental and adult neurotoxicity

There are a number of challenges in developing AOPs for neurotoxicity following neurodevelopmental or adult exposure (Bal-Price et al., 2017). A major concern is a general lack of understanding of the MIEs that are causally responsible for triggering downstream KEs resulting in an AO. Indeed, interplay between the genetic background and exposure to environmental mixtures of chemicals triggers multiple pathways of toxicity complicating the identification of a specific MIE and the subsequent cascade of causatively and linearly linked KEs that result in an AO relevant to the specific symptoms of the disease. Furthermore, the molecular and cellular mechanisms of the patho-physiology of neurodevelopmental or adult neurodegenerative disorders are largely unknown. Additionally, many human neurological disorders may have diverse pathophysiology that underlies similar clinical phenotypes but also conversely, diverse clinical outcomes resulting from similar pathophysiology. For example, Autism Spectrum Disorder (ASD) is a neurodevelopmental disease which is more prevalent in males and that refers to multiple disorders with overlapping clinical symptoms, suggesting that there are shared and unique pathophysiological mechanisms which have yet to be identified (Costa et al., 2017, Lyall et al., 2017, McDonald and Paul, 2010). As a result, the complexity and limited mechanistic understanding of neurological disorders pose challenges in assessing the empirical support (i.e., the pattern of quantitative relationships between KEs up and KEs down) for hypothesized AOPs.

The pathways of toxicity induced by well-known neurotoxicants (e.g., methylmercury, lead, alcohol, polychlorinated bisphenyls, valproic acid, PCBs, POPs, organic solvents etc.) have been studied for many years, illustrating that various pathways of toxicity are triggered by the same chemical, depending on the window of exposure (developing, adult or aging brain) based on the susceptibility of specific brain regions, with different impacts on various neuronal or glial subtypes. As a result, representation of the complexity of disease in in vivo biological systems as a deliberate simplification of a linear series of KEs is necessarily challenging. However, even where information is limited, systematic organization and characterization of the extent of available knowledge of disease process in AOPs based on potentially predictive, causal relationships between the MIE, KEs and AO at the cellular, tissue and organ levels contributes to the identification of critical knowledge gaps in the existing data, as a basis to prioritize additional research.

Despite the challenges mentioned above, several AOPs relevant to central nervous system (CNS) development (Table 1A), as well as the adult and aging brain (Table 1B) have recently been outlined or fully developed (AOP-Wiki, n.d, Bal-Price et al., 2015b, von Stackelberg et al., 2015). These AOPs are mainly qualitative in nature, lacking information sufficient to quantitate KERs. However, the AOPs are well supported based on strong scientific evidence for the biological plausibility of causative links between the MIE, KEs and AOs.

**Table 1A:** Titles of the existing AOPs (fully developed or outlined only) relevant to Developmental Neurotoxicity (DNT).

1.	Chronic binding of antagonist to *N*-methyl-d-aspartate receptors (NMDARs) during brain development induces impairment of learning and memory abilities (AOP-Wiki: https://aopwiki.org/aops/13) (Endorsed by Working Group of the National Coordinators for the Test Guidelines Programme; WNT and Task Force for Hazard Assessment; TFHA)
2.	Chronic binding of antagonist to *N*-methyl-d-aspartate receptors (NMDARs) during brain development leads to neurodegeneration with impairment in learning and memory in aging (AOP-Wiki:https://aopwiki.org/aops/12)
3.	Inhibition of Na +/I − symporter (NIS) decreases TH synthesis leading to learning and memory deficits in children (AOP-Wiki:https://aopwiki.org/aops/54)
4.	Inhibition of Thyroperoxidase and Subsequent Adverse Neurodevelopmental Outcomes in Mammals (AOP-Wiki: https://aopwiki.org/aops/42).
5.	Up-regulation of Thyroid Hormone Catabolism via Activation of Hepatic Nuclear Receptors, Subsequent Adverse Neurodevelopmental Outcomes in Mammals (AOP-Wiki:https://aopwiki.org/aops/8)
6.	Impairment of learning and memory induced by binding of electrophilic chemicals to the SH(thiol)-group of protein and non-protein molecules in neuronal and glial cells during development (Bal-Price, Crofton, Sachana, et al., 2015)
7.	The interaction of non-dioxin-like PCBs with ryanodine receptors causes their sensitization affecting neuronal connectivity that results in behavioural deficits (developmental neurotoxicity) (Bal-Price, Crofton, Sachana, et al., 2015)
8.	Exposure to Mixtures of Metals and Neurodevelopmental Outcomes: A Multidisciplinary Review Using an Adverse Outcome Pathway Framework (von Stackelberg et al., 2015)

**Table 1B:** Titles of the AOPs (fully developed or outlined only) relevant to Neurotoxicity (NT).

1.	Binding of agonists to ionotropic glutamate receptors in adult brain causes excitotoxicity that mediates neuronal cell death, contributing to learning and memory impairment (endorsed by WNT and TFHA) (AOP-Wiki: https://aopwiki.org/aops/48)
2.	Binding to the picrotoxin site of ionotropic GABA receptors leading to epileptic seizures (AOP-Wiki: https://aopwiki.org/aops/10)
3.	Inhibition of the mitochondrial complex I of nigra-striatal neurons leads to parkinsonian motor deficits (AOP-Wiki: https://aopwiki.org/aops/3)
4.	Binding to SH/seleno-proteins can trigger neuroinflammation leading to neurodegeneration (AOP-Wiki:https://aopwiki.org/aops/17)
5.	Binding of pyrethroids to voltage-gated sodium channels induces acute neurotoxicity (Bal-Price, Crofton, Sachana, et al., 2015)
6.	Binding of organophosphates to neuropathy target esterase (NTE) results in delayed (Bal-Price, Crofton, Sachana, et al., 2015)
7.	Multiple molecular initiating events trigger neuro-inflammation leading to neurodegeneration (Bal-Price, Crofton, Sachana, et al., 2015)
8.	The interaction of redox cycling chemicals with NADH cytochrome *b*5 reductase and NADH-quinone oxidoreductase results in NAD^+^ formation causing reduced adult neurogenesis (Bal-Price, Crofton, Sachana, et al., 2015)

While developed by toxicologists to facilitate transition to more predictive approaches for the assessment of the hazard of chemicals and drugs, AOP concept is also a useful tool to capture complex biological knowledge on known disease pathways as illustrated below by two examples of AOPs, describing learning and memory deficit in children and parkinsonian motor deficit in PD as adverse outcomes of relevant AOPs.

In general, AOP development for a disease is facilitated by available experimental models of pathogenesis known to be induced by toxicant(s) and knowledge on functional and structural relationships for different stages of the disease progression which can be blocked experimentally. The availability of non-invasive biomarkers or measures of different steps in the pathogenesis of the disease also contributes to the development of AOPs.

## 5 AOP relevant to developmental neurotoxicity (DNT): chronic binding of antagonist to *N*-methyl-d-aspartate receptors (NMDARs) during brain development induces impairment of learning and memory abilities

CNS is one of the most complex organs in the body, consisting of different subtypes of neurons and glia, varying structural characteristics (e.g., size, spatial configurations), synaptic functions (inhibitory, excitatory), neurotransmitters release (e.g., acetylcholine, glutamate, GABA) and possessing high energy requirements (vesicular release of neurotransmitters, ion channels activity, action potentials etc.). The specialized metabolic, morphological and physiological features of the CNS result in unique vulnerabilities to toxic compounds that may act on multiple sites through different toxicity pathways.

It is well documented and understood that the developing nervous system is more vulnerable than the adult CNS to chemical exposure due to complex developmental processes such as the commitment and differentiation of the neuronal progenitor cells followed by glial and neuronal cell proliferation, migration, differentiation into various neuronal and glial subtypes, synaptogenesis, pruning, myelination, networking and terminal functional neuronal and glial maturation (Bal-Price et al., 2012, Fritsche et al., 2017, Hogberg et al., 2010, Hogberg et al., 2009, Krug et al., 2013, Rice and Barone, 2000, Yang et al., 2014). Moreover, the incompletely formed blood brain barrier (BBB) may facilitate chemical uptake within the foetus/neonatal brain (Adinolfi, 1985, Rodier, 1995). The neurodevelopmental outcome depends not only on the nature of chemical exposure (dose, duration) but also on the stage of brain development (cells proliferation, migration, differentiation or maturation) at the time of exposure (Rice & Barone, 2000).

Results of epidemiological studies indicate that environmental chemicals contribute to the observed increase of neurobehavioral disorders in children (Grandjean & Landrigan, 2014) including lowered IQ, learning disabilities, attention deficit hyperactivity disorder (ADHD) and, in particular, autism (Kalkbrenner, Schmidt, & Penlesky, 2014). This highlights the pressing need for new methodologies that can more rapidly and cost-effectively screen large numbers of chemicals for their potential to cause neurodevelopmental toxicity, including cognitive damage in children and to undertake actions to control or eliminate relevant exposures in the environment (Bal-Price et al., 2015b, Fritsche et al., 2017).

Recently, an AOP has been developed according to the OECD guidance (OECD, 2013) documenting the causative link between the blockage of *N*-methyl-d-aspartate (NMDA) receptors during brain development (particularly synaptogenesis) and learning and memory impairment based on data obtained after exposure to lead as reference chemical (AOP-Wiki; AOP 13 https://aopwiki.org/aops/13). This AOP has been independently scientifically peer-reviewed as part of the OECD program, endorsed by the Task Force for Hazard Assessment (TFHA) and Working Group of the National Coordinators for the Test Guidelines Programme (WNT) and subsequently published in a dedicated series (OECD, 2016a).

Based on epidemiological studies of children (Baghurst et al., 1992, Bellinger, 2004, Needleman and Gatsonis, 1990), as well as experimental studies in vivo (Anderson, Pothakos, & Schneider, 2012) and in vitro (Neal et al., 2010, Neal et al., 2011, Stansfield et al., 2012), it is well documented that lead (Pb^2 +^) exposure (which blocks the NMDA receptor) during brain development impairs a variety of cognitive, behavioural and neurochemical processes resulting in deficits in learning, memory, attention, impulsivity and executive function (Needleman and Gatsonis, 1990, Sanders et al., 2009). Exposure to Pb^2 +^ during early development in epidemiological studies (Baghurst et al., 1992, Bellinger, 2004, Canfield et al., 2003, Finkelstein et al., 1998, Lanphear et al., 2005, Needleman and Gatsonis, 1990, Surkan et al., 2007) and experimental animals, results in long-term behavioural abnormalities and cognitive deficits (Moreira et al., 2001, Murphy and Regan, 1999). Multiple lines of evidence indicate that Pb^2 +^, even in low concentrations, can impair hippocampus-mediated learning processes in animal models (reviewed in (Toscano & Guilarte, 2005)). There is also evidence that the non-competitive antagonist of the NMDA receptor, MK-801, induces dose-dependent impairment of learning and memory in animals (van der Staay et al., 2011, Wong et al., 1986), including nonhuman primates (Ogura & Aigner, 1993) as confirmed in human studies using NMDA antagonists such as ketamine and phencyclidine (reviewed in (Rezvani, 2006)). The experimental results supporting the essentiality data for each KE described in this AOP are discussed in detail in the AOP-Wiki (AOP 13: https://aopwiki.org/aops/13).

**Fig. 3:**
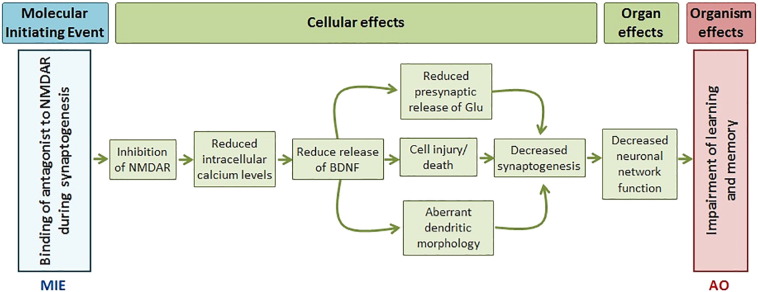
Graphical representation of the key events at the cellular, organ and organism level triggered by binding of an antagonist to the NMDA receptor (MIE) and resulting in the adverse outcome (AO), defined as impairment of learning and memory.

Based on a review of the available data, the MIE in this AOP was defined as the chronic binding of an antagonist to NMDA receptors during synaptogenesis (process of synapse formation). Resulting inhibition of NMDA receptor function triggers the sequence of the following KEs at different biological levels: the reduction of intracellular calcium levels, a decrease of BDNF levels, reduced presynaptic glutamate release, aberrant dendritic morphology, increased cell death, decreased synaptogenesis resulting in decreased neuronal network function, causing learning and memory deficit (AO) (Fig. 3). Relationships between the KEs are based on strong supporting evidence on biological plausibility as described in detail in the AOP Wiki (AOP 13; https://aopwiki.org/aops/13).

It is well documented in the existing literature that learning and memory processes rely on physiological functioning of the NMDA receptors which have been demonstrated in both animal and human studies investigating the effects of NMDA receptor antagonists and in mutant mice lacking NMDA receptor subunits (reviewed in (Granger et al., 2011, Haberny et al., 2002, Rezvani, 2006)). However, the initial interaction between a chemical and molecular, cellular target that triggers the cascade of key events causatively linked to impairment of learning and memory in children (adverse outcome) could be triggered by multiple MIEs, including the blockage of NMDA receptor function during synaptogenesis.

As mentioned above, by definition, AOPs are chemical agnostic though their documentation relies on supporting evidence of effects in animals and humans of chemicals or other stressors which initiate the cascade of events (Villeneuve et al., 2014a, Villeneuve et al., 2014b). For this AOP, the empirical support is based principally on experimental data obtained after exposure to lead (reference chemical) since there are abundant in vivo and in vitro experimental data and epidemiological studies strongly suggesting that cognitive deficit is linked to chronic exposure to this heavy metal. However, in principle, any chemicals/drugs that will inhibit the activity of the NMDA receptor during brain synaptogenesis (in humans from the third trimester of pregnancy until 2–3 years following birth) (Bai, Twaroski, & Bosnjak, 2013) could potentially trigger the sequence of cellular KEs defined in the AOP (Fig. 3) resulting in a decreased number of synaptic connections, and altered neuronal network formation and function leading to cognitive impairment in children (AO).

As mentioned earlier, by definition AOPs are pragmatic simplifications of complex biology, focused on the critical, measurable KEs at different biological organization level (cellular, tissue, organ) leading to AO. Taking into consideration the complexity of cognitive function, the proposed MIE and sequence of defined KEs that result in learning and memory deficit is one of many possible cascades of pathways leading to the same AO. The further development and assembly of AOPs into networks through close collaboration of toxicologists and clinicians, could contribute to documentation of the complexity of this AO (learning and memory impairment) which is likely triggered by variety of MIEs and a range of different pathways. Network of AOPs, based on systematic documentation of existing and evolving biological knowledge, could guide the development of testing strategies using in vitro assays anchored to AOP KEs or more reliably common KEs identified in AOPs network (Fig. 5), enabling efficient screening of large number of chemicals to identify those with potential to cause cognitive impairment in children.

## 6 AOP relevant to neurodegenerative disorder: inhibition of the mitochondrial complex I of nigra-striatal neurons leads to parkinsonian motor deficits

Parkinson's disease (PD) is a late-onset, progressive neurodegenerative disorder characterized by relatively selective nigrostriatal dopaminergic degeneration and the development of fibrillary cytoplasmic inclusions (Lewy bodies) containing aberrant oligomeric alpha-synuclein and ubiquitin (Bove and Perier, 2012, Fujita et al., 2014, Schapira, 2006). The aetiology of PD is not completely understood, but it is believed to be caused by the interaction of a range of genetic and environmental factors (e.g. exposure to pesticides such as rotenone, paraquat and dichlorodiphenyltrichloroethane; DDT) (Schapira, 2006, Sherer et al., 2002, Song and Cortopassi, 2015, Tanner et al., 2011, Zaltieri et al., 2015, Zhu and Chu, 2010). It appears to involve multiple interacting pathways including mitochondrial dysfunction (Hoglinger et al., 2003, Ikawa et al., 2011, Lin and Beal, 2006), impaired protein degradation (Chen et al., 2007, Cherra et al., 2010, Domingues et al., 2008, Esteves et al., 2011, Fornai et al., 2005), α-synuclein pathobiology (Lee et al., 2002, Lotharius and Brundin, 2002, Saha et al., 2004), neuroinflammation (Iannaccone et al., 2013, Mena and Garcia de Yebenes, 2008, Verkhratsky et al., 2014), resulting in selective dopaminergic (DA) neuronal cell death in the substantia nigra pars compacta (SNpc), finally leading to motor deficit (Bernheimer et al., 1973, Calne and Sandler, 1970, Fujita et al., 2014, Koller, 1992, Leenders et al., 1986, Snow et al., 2000). Definition of a specific MIE that triggers a cascade of these cellular and tissue KEs in PD pathogenesis, linked to each other in a causative manner resulting in specific symptoms, characteristic of PD pathogenesis is complicated by the interconnections between deregulated molecular and cellular pathways (Fujita et al., 2014).

Some PD cases may have a clear genetic background while others could be idiopathic, caused by toxins (e.g., MPTP) (Fornai et al., 2005, Thomas et al., 2012) or the interplay of both these factors. Despite different aetiology, all PD cases are characterized by the loss of DA neurons projecting from the SNpc to the putamen (Calne and Sandler, 1970, Koller, 1992, Snow et al., 2000, Sulzer, 2007). Multiple epidemiological studies suggest that exposure to pesticides (e.g., organochlorine insecticides such as DDT, paraquat and rotenone) may present a potentially important environmental risk factor for developing PD (e.g., (Ntzani, Chondrogiorgi, Ntritsos, Evangelou, & Tzoulaki, 2013). However, the causative links between the exposure to a specific environmental chemical and the mechanisms underlying PD pathogenesis are not fully understood.

The EFSA (European Food Safety Agency) PPR (Plant Protection Products and their Residues) Panel Working Group applied the AOP conceptual framework to determine whether the association between exposure to pesticides and PD observed in a meta-analysis of epidemiological studies (Ntzani et al., 2013) could be supported by biological plausibility and mechanistic understanding of pathways involved.

Based on a systematic literature review, the EFSA PPR Panel Working Group has developed an AOP (AOP-Wiki) entitled “*Inhibition of the mitochondrial complex I of nigra-striatal neurons leads to parkinsonian motor deficits*” following the OECD guidance (OECD, 2013) and accompanying Handbook (OECD, 2016b). The MIE has been defined as binding of an inhibitor to complex I (CI) of the mitochondrial respiratory chain which results in the inhibition of CI function, triggering a sequence of KEs including mitochondrial dysfunction, impairment of cellular proteostasis processes, neuro-inflammation, leading to the selective degeneration of DA neurons in SNpc and resulting in the parkinsonian motor deficit symptoms described in this AOP as the AO. The supporting evidence for the essentiality of the identified KEs in this AOP is considered high as knock out animal models, engineered cells or replacement therapies globally demonstrate that blocking or attenuating KEs up prevents KE down or the AO (AOP-Wiki: https://aopwiki.org/aops/3).

**Fig. 4:**
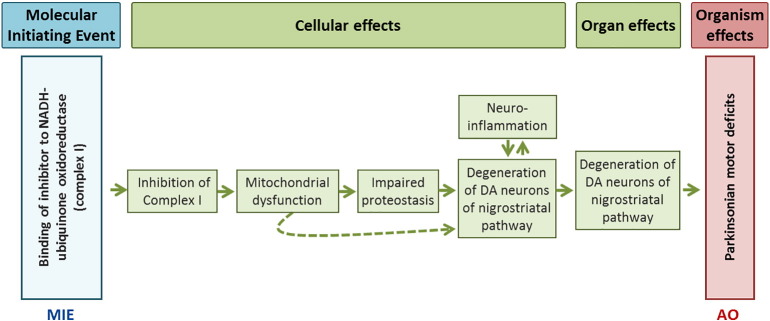
Schematically represented molecular initiating event (MIE), key events identified at different biological levels and adverse outcome (AO) of the AOP entitled: Inhibition of the mitochondrial complex I of nigra-striatal neurons leads to parkinsonian motor deficits.

Moreover, data from in vitro and in vivo experiments are complemented by human studies where the presence of KEs and associated KERs (*Empirical support for linkage* in KER (AOP-Wiki: https://aopwiki.org/aops/3) have been identified in the post mortem brain tissue from individuals with sporadic PD. All these data support the biological plausibility of described KERs in this AOP (Fig. 4). For instance there are several clinical studies describing impairment of catalytic activity of CI (Parker & Swerdlow, 1998), presence of striatal oxidative stress that is linked to the progression of disease severity) (Ikawa et al., 2011), presence of aggregated, poly-ubiquitinated proteins in Lewy Bodies (Betarbet, Sherer, & Greenamyre, 2005), failure of ubiquitine-proteasome system (McNaught and Olanow, 2003, McNaught et al., 2001), confirming impairment of proteasomal activity. Furthermore, correlation between striatal dopamine loss and degeneration of DA neurons in SNpc, accompanied by inflammation that results in motor deficit has been strongly documented (see *Empirical support* for each KER described in https://aopwiki.org/aops/3).

The overall weight of evidence indicates a strong biological plausibility between the inhibition of mitochondrial complex I (MIE) and parkinsonian motor deficit symptoms (AO) through the described AOP and causatively linked KEs at the cellular and organ level (Fig. 4). Empirical support for the KERs of this AOP is based on clinical data, as well as *in vivo* and *in vitro* experiments following exposure to two reference chemicals, rotenone (pesticide) and 1-methyl-4-phenyl-3104 1,2,3,6-tetrahydropyridine (MPTP). The toxicological data available after exposure to rotenone or MPTP for empirical support of the identified KERs (described in detail in AOP-Wiki, https://aopwiki.org/aops/3) suggest that both chemicals were able to reproduce *in vivo* and/or *in vitro* some cellular and tissue features of PD leading to motor impairment.

Clinical studies have shown that exposure to MPTP in humans and non-human primates (experimental data) produced Parkinson-like motor deficit after only a few days of exposure (Bernardi, 1999, Burns et al., 1985, Langston et al., 1984, Lee et al., 2011, Siegel et al., 1999). MPTP crosses the blood-brain barrier and is selectively taken up by DA transporters of DA neurons after metabolic activation to MPP^+^ by MAO-B (mono-amino-oxidase B) in astrocytes (Cleeter et al., 1992, Greenamyre et al., 2001). Rotenone is a highly lipophilic insecticide/pesticide which, unlike MPP^+^, lacks specificity for DA neurons transporters but causes characteristic features of PD when chronically administered to rodents at low doses (Betarbet et al., 2000, Ojha et al., 2015, Sanders and Greenamyre, 2013, Sherer et al., 2002, Sherer et al., 2003). The adverse outcome (parkinsonian motor deficit) is considered here as consequence of the inhibition of CI of the mitochondrial respiratory transport chain triggered by exposure to rotenone and MPTP. However, any pesticides or other types of chemicals that inhibit CI function (MIE) resulting in parkinsonian motor deficit symptoms (AO) will be relevant to this AOP as well.

Interestingly, both MPP^+^ and rotenone can produce neurotoxicity also by other mechanistic pathways, not only through inhibition of CI function (Segura-Aguilar & Kostrzewa, 2015).

This single AOP does not represent the complexity of PD but describes one of many possible cascade of events leading to AO defined here as parkinsonian motor deficit and does not address other symptoms of PD (dementia, sleep disturbance, emotional problems, etc.). Hopefully, multiple AOPs will be developed soon, with MIEs that are causally linked to other PD symptoms. Linking of multiple single AOPs, for different symptoms of PD into AOPs network will more realistically represent the biological complexity of PD pathophysiology, permitting better understanding of interacting pathways implicated in PD, possibly facilitating targeted treatment of early PD symptoms, referring to early KEs identified in the relevant AOPs.

Discovering causal factors of PD pathogenesis by identification of MIEs is difficult because pathways deregulated in neurodegeneration are interconnected and influence each other. Therefore illustration of interactions across different pathways within AOPs network or PD interaction map (Fujita et al., 2014) may help to identify key factors in PD pathology.

## 7 AOPs applications for neurotoxicity testing

**Fig. 5:**
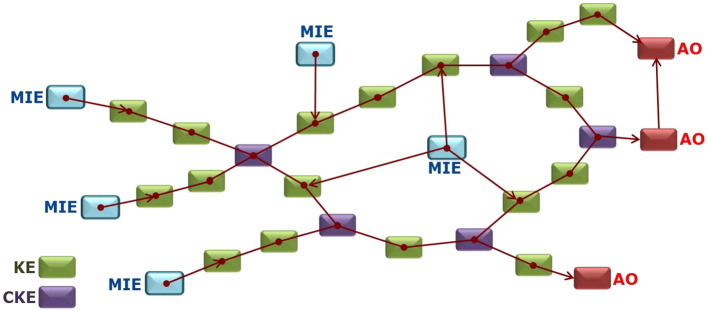
Multiple AOPs sharing common KEs (CKE) (nodes) can be assembled into networks. AOP networks more realistically capture the complexity of biological systems where single molecular initiating events (MIE) can trigger multiple adverse outcomes and multiple MIEs can lead through a cascade of key events (KE) to the same adverse outcome (AO). Development of additional AOPs contributes to the description of such networks.

Single AOPs constitute deliberate simplification of complex biological systems intended to support regulatory decision making as a basis to integrate information from various levels of biological organization (cellular, tissue, organ) to identify critical uncertainties and knowledge gaps, guiding additional experimental work. While individual AOPs (i.e., a sequential cascade of KEs leading to adverse outcome) are likely to be triggered by a limited number of neurotoxic compounds, assembly of single AOPs into networks (Fig. 5) through interconnected pathways and identification of common key events in several AOPs (Bal-Price, Crofton, Sachana, et al., 2015), are likely to represent more realistic descriptions of the complexity of disease pathophysiology. Assembled AOPs network(s) reflect more realistically that single MIE can trigger multiple AOs and that multiple MIEs can lead to the same AO (Fig. 5). The numbers of AOPs currently available are limited. However, their contribution to comprehensive and holistic networks will undoubtedly contribute to the improvement of mechanistic understanding of pathways interactions involved in various neurological disorders. AOP networks are also envisaged to facilitate neurotoxicity testing of combined exposures to multiple chemicals, illustrating interplay between diverse involved pathways of toxicity triggered by a range of MIEs, representing exposure to mixture of chemicals.

Mechanistic knowledge incorporated in existing AOPs relevant to DNT or NT is sufficient, currently, to more effectively guide the selection of in vitro assays for neurotoxicity testing, particularly when anchored to the identified MIEs and common KEs (Bal-Price et al., 2015a, Bal-Price et al., 2015b). Such a battery of in vitro assays has potential to form the basis of more predictive AOP-informed Integrated Approaches to Testing and Assessment (IATA) development (Fig. 2) for hazard characterization in risk assessment (Worth & Patlewicz, 2016) of potential neurotoxicants. Data from batteries of assays included in an IATA will facilitate building of prediction models in the application of defined approaches to testing and assessment. In defined approaches, (mechanistic) data generated by non-animal methods (i.e. in silico, in chemico, in vitro) and animal models, deemed to be fit-for-purpose are evaluated by means of a fixed data interpretation procedure (DIP), excluding expert judgment evaluation. The results coming from the fixed DIP can serve as a base to build an algorithm for a prediction model, establishing a threshold (a concentration and time of exposure to a chemical) for triggering neurotoxicity.

It should be noted that mechanistic information described in AOPs and MOAs relevant to neurotoxicity, facilitates its application not only for priority setting and assessment of hazard for chemicals (single or in mixture) but has potential to contribute also in early safety assessment in the drug discovery process (Edwards et al., 2016, Friend and Schadt, 2014, Schadt and Bjorkegren, 2012) using the same testing strategies.

Identified MIEs and KEs could serve as a basis for developing quantitative structure-activity relationships (QSAR) and chemical structure-based read-across models that can be applied for screening of chemicals to identify those that have potential to trigger disease/neurotoxicity. Taking an example of the DNT AOP, QSARs could be developed for the MIE to identify chemicals/drugs for which structure would indicate possible binding and blockage of the NMDA receptor function, facilitating screening and prioritization of chemicals or drugs with potential to cause learning and memory deficit in children (Fig. 3).

Recently, such a QSAR model for non-competitive antagonists of NMDA receptor has been developed based on relationships for 48 substituted MK-801 derivatives (Chtitaa, Larifb, Ghamalia, Bouachrinec, & Lakhlifia, 2015) quantitated through multivariate statistical analyses. Chemicals with potential to inhibit NMDA receptor function could be further evaluated to verify whether they are able to trigger a cascade of key events described in the relevant AOPs (Table 1A, Table 1B) including AOP on *Chronic binding of antagonist to N-methyl-d-aspartate receptors (NMDARs) during brain development induces impairment of learning and memory abilities* (https://aopwiki.org/aops/13). In vitro assays anchored to the identified KEs within this AOP would permit evaluation of intracellular calcium and brain derived neurotrophic factor (BDNF) levels, neuronal differentiation, synaptogenesis, and neuronal network formation and function. These in vitro assays have been well standardized not only in animal (Bal-Price et al., 2012, Harrill et al., 2015, Mundy et al., 2008) but also human mixed neuronal/glial cultures derived from human embryonic or induced pluripotent stem cells (hiPSCs) (Baumann et al., 2014, Pamies et al., 2016, Zagoura et al., 2016). In vitro methods based on human (rather than animal) cells may contribute to a better understanding of biology and disease process in humans. Indeed, it is currently possible to generate hiPSCs from donated somatic cells and differentiate them into disease- and patient-specific neural cells. Neuronal in vitro models derived from hiPSCs are able to express biomarkers of disease for AD (amyloid precursor protein, beta- and gamma- secretase and accumulation of amyloid) (Yahata et al., 2011) and PD (mutations in PINK1), being useful for drug screening (Azkona et al., 2016, Yahata et al., 2011).

As illustrated by the development of AOP for parkinsonian motor deficit (AO), consideration of available mechanistic data within the organizational construct of the AOP may also contribute to the assessment of biological plausibility in support for (or against) causality of observed associations in epidemiological studies.

In vitro assays developed for the KEs within described AOPs relevant to neurotoxicity may also contribute to better quantitative characterization of the predictive relationships concerning the degree of perturbation required for KEs up (concentration and time of exposure) to trigger KEs down. Such data could serve as a basis for the development of computational models permitting quantitative translation of information on earlier KERs into predictive probability or severity of the AO.

The identified KEs could also serve as a tool for grouping of chemicals according to their biological activities, permitting identification of those that despite different chemical structure, trigger the same KE (biological grouping). This would increase confidence in grouping of chemicals according to their biological activity into categories according to their triggered MIEs or KEs. Therefore, AOPs provide a strong pathophysiological rationale to compound classification, having an added value for DNT testing as complex nature of the underlying pathways of toxicity is inadequately captured by current chemical category formation solely based on chemical structure or physical-chemical properties.

In summary, testing based on in vitro assays anchored to common KEs of AOP network(s) has potential to increase identification of neurotoxicants, triggered by different MIEs, mediated through various pathways and resulting in diverse AOs, suitable for screening and prioritization of chemicals (or drugs) for further testing.

## 8 Moving towards mechanistically based taxonomies of human disease using AOP concept

The adverse outcome pathway concept, originally developed for regulatory toxicology as illustrated through examples in this review, provides the description and basis for potential modelling of physiological cellular signaling pathways which once sufficiently perturbed (e.g., by exposure to a chemical), become patho-physiological pathways leading to disease (adverse outcome). Adverse outcomes in the AOPs included here (e.g., PD) are reported symptoms of neurological disease.

Human diseases are currently classified based on clinical features (http://www.who.int/classifications/icd/en/), rather than their underlying patho-physiological pathways responsible for different symptoms of the same disease. Mechanistic understanding of these pathways described in AOP format for various symptoms of a disease as causatively linked KEs at the cellular, tissue and organ level could contribute to the establishment of more “mechanistically-based” taxonomies of disease. Such knowledge could potentially lead to the re-classification of certain pathological states according to their underlying patho-physiological pathways facilitating the identification and development of well-targeted individual (personalized) treatment of the various symptoms of the disease, especially if investigated in hiPSCs-derived neural cells originated from patients (Yahata et al., 2011). Development of human- and disease-specific models and tools would avoid limitations of animals studies related to species differences (Langley, et al., 2017). Indeed, it is widely recognized that animal models recapitulate only limited pathological mechanisms of human disease (Tsukamoto, 2016) necessitating additional focus on human-relevant models (NRC, 2007).

AOP-based descriptions are consistent, then, with the objective proposed by Kola and Bell (2011) “to reform the taxonomy of human disease” towards mechanism based-classifications. These authors claim that the lack of recognition of disease heterogeneity reduces the likelihood of success of clinical trials. Indeed, currently, more than 90% of new compounds entering clinical trials fail due to insufficient efficacy and poor evaluation of toxicity side effects (Plenge, Scolnick, & Altshuler, 2013).

Transition to more mechanistically-based disease classifications would be consistent with the envisaged paradigm shift in toxicity testing, moving away from adverse effects evaluation in whole-animal models to human relevant in vitro methods, measuring early biomarkers, predictive of late adverse outcome. For example, for the “disease” AOPs described here, early KEs could be quantitatively measured by in vitro test methods using hiPSCs-derived neural cells originated from PD patients or children suffering from decreased learning and memory abilities. The potential of patient-derived iPSCs differentiated into neural cells has been illustrated in a wide range of experimental investigations including in vitro developmental neurotoxicity testing (Schwartz et al., 2015), the modelling of neurological disorders (Russo, Cugola, Fernandes, Pignatari, & Beltrao-Braga, 2015), AD-associated gene regulatory networks, Rett syndrome (a severe form of autism spectrum disorders) (Marchetto et al., 2010) and drug-screening (Beltrao-Braga & Muotri, 2017).

The AOP concept, though developed to contribute to the transition to more efficient and effective toxicity testing has potential, then to contribute also in the biomedical field. Mechanistic understanding of the pathways deregulation that underlies the transition from a “normal physiological state” to disease state could be fundamental for discovery of new approaches to either prevent disease or to interfere with disease symptoms (Hofmann-Apitius, Alarcon-Riquelme, Chamberlain, & McHale, 2015).

Based on developed AOPs, for example, mechanistically defined subgroups of PD patients with different symptoms (supported by understanding of underlying pathways) could be identified, leading to the new taxonomy of PD diseases. MIEs and KEs described in these AOPs could serve as potential drug targets, improving disease diagnoses, personalized treatment and potential disease prevention.

The development of mechanistically-based taxonomies of diseases is being investigated in the ongoing projects PRECISESADS (http://www.preciesads.eu) and AETIONOMY (http://www.aetionomy.eu) for autoimmune disorders and neurodegeneration, respectively. Similar approaches have been investigated for AD where signaling pathways and their corresponding anatomical regions in the human brain for Alzheimer's have been mapped increasing understanding of the mode-of-action of the approved drug Rasagiline (Iyappan et al., 2016).

Along this line, novel human-based cellular and computational models, together with epidemiological and clinical studies may facilitate the acquisition of human-relevant data to improve mechanistic understanding of AD pathology, as recently advocated (Pistollato et al., 2015, Pistollato et al., 2016). Similarly, a comprehensive molecular interaction map that integrates pathways implicated in PD has been created (Fujita et al., 2014).

Application of the AOP concept as a starting point for the development of “mechanistically-based” taxonomies of diseases has the potential to contribute, then, to increasing confidence in drug target selection, potentially improving success rates in discovery and development.

## 9 Conclusions and future directions

The AOP framework provides a simple construct to integrate data at various levels of biological organization to contribute, along with other initiatives, in the transition of toxicity testing to more predictive approaches, necessitated by increasingly demanding regulatory mandates to assess hazard associated with large numbers of environmental chemicals.

The systematic documentation and assessment of mechanistic understanding of pathways described as AOPs are anticipated to contribute to a number of applications, including chemical biological grouping or categorization, development of QSARs for chemicals that might trigger MIEs and the development of mechanistically informed IATA. The development of in vitro assays for MIEs and early KEs in described AOPs has potential to facilitate development of more tailored, purpose-specific AOP-informed IATA establishment, increasing the efficiency of testing for different regulatory purposes.

Examples of AOPs describing the pathways to specific symptoms (AO) of developmental neurotoxicity (impairment of learning and memory in children) and PD (parkinsonian motor deficit) described here illustrate that the AOP concept has potential to contribute beyond regulatory toxicity testing of chemicals in health research, including drug discovery and drug toxicity testing through the advancement, of human-cell based in vitro test methods to improve the mechanistic understanding of human disease pathways that often cannot be investigated in animal models.

In summary, the systematic description of disease pathways as interconnected networks of AOPs for various disease symptoms could contribute to the reclassification of certain pathological states based on current mechanistic understanding, resulting possibly in more personalized treatment. To achieve this, closer collaboration of clinicians and toxicologists is required to develop critical numbers of AOPs, permitting AOPs network descriptions to better represent existing knowledge on underlying mechanisms of disease pathways.

## Conflict of interest

The authors declare that there are no conflicts of interest.
